# Assessment of Commercially Available Polyethylene Recyclates for Blow Molding Applications by a Novel Environmental Stress Cracking Method

**DOI:** 10.3390/polym15010046

**Published:** 2022-12-22

**Authors:** Paul J. Freudenthaler, Joerg Fischer, Reinhold W. Lang

**Affiliations:** Institute of Polymeric Materials and Testing, Johannes Kepler University Linz, Altenberger Straße 69, 4040 Linz, Austria

**Keywords:** blow molding, polyethylene, environmental stress cracking resistance, cracked round bar specimens, recyclate, fatigue, slow crack growth

## Abstract

The transition to a circular economy has a major impact on waste management and the reuse of materials. New mandatory recycling targets for plastics will lead to a high availability of recyclates. For these recyclates, useful applications need to be found. One potential application for recyclates is blow molding bottles as packaging for non-food contents. This study investigates commercially available post-consumer high-density polyethylene recyclates together with virgin blow molding grades in terms of their short-term mechanical properties and environmental stress cracking resistance. While the short-term mechanical properties showed only slightly lower performance than the tested virgin grades, the overall environmental stress cracking failure times of the recyclates were much lower compared to virgin materials, even though the crack-growth kinetics could be similar. Although neither the tensile nor the notched impact strength results of the two polyethylene recyclates revealed large differences, the stress intensity-factor-dependent crack-growth rates of both materials were significantly different.

## 1. Introduction

The current endeavors of the European Commission towards a circular economy have led to several regulations concerning packaging products [1,2]. The recycling target of 50% by the end of 2025 and 55% by the end of 2030 [3] for plastic packaging waste is an ambitious goal, especially considering the currently low recycling rates for the biggest group of packaging plastics, polyolefins. One of the major packaging materials [4], high-density polyethylene (PE-HD), makes up 17% of the plastic waste [5], but only a small part is recycled in Europe [6]. One of the main characteristics of PE-HD which led to its high usage as a packaging material is its high impact toughness [7]. Popular examples of PE-HD packaging products are bottles, bottle caps, canisters, and blow-molded cosmetics packaging such as shampoo bottles, etc., [8].

Polyolefin waste is notorious for its difficulty of separation due to the overlapping densities of the various polyolefins [5,9]. Thus, polyolefin recyclates exhibit varying purity and performance [10], which depend on the feedstock, sorting and reprocessing. Several companies offer recycled high-density polyethylene (rPE-HD) with various quality and/or purity levels. This study aims to evaluate the different commercially available rPE-HD grades for their usage as an extrusion blow molding material in comparison to the relevant virgin material grades in terms of their mechanical properties.

Virgin blow molding PE-HD grades have melt mass-flow rates (MFRs) of between 0.25 g/10 min and 0.80 g/10 min (according to ISO 1133-1 [11] at 190 °C and 2.16 kg) [12,13,14,15]. Furthermore, the promoted properties in the relevant material datasheets include yield strengths between 20 MPa and 32 MPa, tensile moduli between 1000 MPa and 1420 MPa (both according to ISO 527-1 [16] and ISO 527-2 [17]), high-impact strengths tested with different testing methods at various temperatures, and an otherwise seldomly used property: environmental stress cracking resistance (ESCR), also tested with various methods. ESCR is a material property, which is specifically needed for the containment of crack-growth accelerating liquids (e.g., detergents) and is highly dependent on the specimen type, loading conditions, and used environment (media and temperature).

The post-consumer recyclate market offers a wide range of rPE-HD grades in varying colors. The most desirable color for most applications is natural (without color additive) followed by white. Both colors are used for natural or white products, respectively, or for subsequent coloration. Some suppliers offer color separated recyclate grades. Due to the lack of color separation and thus the color mixing of differently colored flakes, the most common colors on the post-consumer recyclate market are shades of grey. Commercially available rPE-HDs from post-consumer packaging waste usually have higher MFRs, as they consist of different sources of PE waste and include injection-molded products, for instance, caps and closures. While the MFR, tensile, and impact properties can typically be found in the datasheets of rPE-HDs, ESCR is a rarely declared property.

Several standardized test methods for the experimental characterization of the ESCR of PE-HD are in use. These test methods make use of accelerating media and/or test temperatures to shorten testing times. A widely used test method, the full-notch creep test (ISO 16770) [18], uses elevated temperatures (50 °C–90 °C) and a creep crack-growth accelerating mixture of deionized water and surfactant. Other ESCR methods, such as ASTM D 1693 [19] or ASTM D 2561 [20], also use elevated temperatures and surfactants. The cracked round bar (CRB) test method according to ISO 18489 [21], a method initially introduced to characterize the resistance against slow crack growth in PE pipe grades, uses neither elevated temperature nor harsh media, but cyclic loading for the acceleration of crack growth and shortening of testing times. As the authors have experience with superimposed cracked round bar experiments [22], the method is well-established in the field of fracture mechanics [23,24,25,26], and as this method allows for the in situ measurement of the crack-growth rate, this testing method was chosen for comparison of the ESCR of virgin and recycled PE-HD grades. The measurement of the crack length enables the determination of the crack-growth rate which offers more insight into the fracture mechanical failure than failure times alone.

## 2. Materials

**Figure 1:**
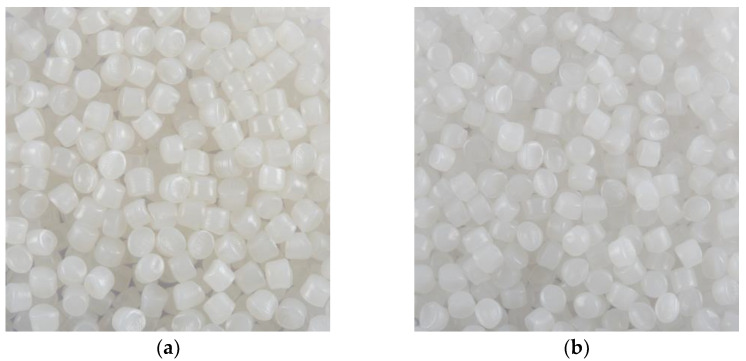
Images of the pellets of the virgin blow molding PE-HD grades vPE-1 (**a**) and vPE-2 (**b**).

Two virgin blow molding PE-HD grades in the form of pellets, as can be seen in Figure 1, were acquired as benchmark grades for this study. Both PE-HD grades were translucent without color/white pigment. The only apparent optical difference was the yellow tint of vPE-1, as can be seen in Figure 1a in comparison to Figure 1b.

**Figure 2:**
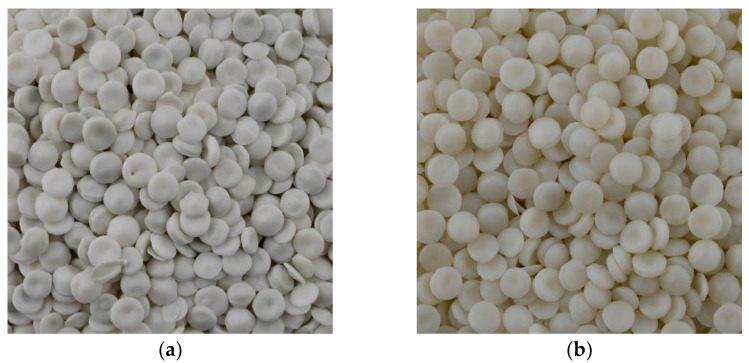
Images of the pellets of the PE-HD recyclates rPE-A in opaque white (**a**) and rPE-B in natural (**b**).

Considering the two basic constraints, color and MFR, two rPE-HD grades were purchased from two different European suppliers based on their white (rPE-A) and natural (rPE-B) colors and MFRs, which are in the range of the benchmark virgin grades. The recyclates were delivered as pellets, as can be seen in Figure 2. rPE-A is an opaque white color while rPE-B has a more translucent tone or rather natural color.

## 3. Methods

Differences in specimen preparation, conditioning, and testing often impede objective comparisons between material data of different suppliers. Moreover, small lot-to-lot variations can occur even for virgin grades. Therefore, within the scope of this study, all basic properties were characterized by identical parameters for better comparability and accurate structure–property–performance relationships.

### 3.1. Melt Mass-Flow Rate Measurements

The MFR measurements were conducted at 190 °C and with both 2.16 kg and 5 kg static loads on the melt flow indexer Mflow (ZwickRoell GmbH & Co. KG, Ulm, Germany) according to ISO 1133-1 [11] and ISO 17855-2 [27]. Cuts were automatically made after every 1 mm (2.16 kg) and 3 mm (5 kg) piston movement. The time between cuts was measured and each extrudate was weighed on an ABS 220-4 electronic balance (Kern & Sohn GmbH, Balingen-Frommern, Germany). The MFR in g/10 min was calculated by extrapolation to 10 min for the individual cut. For each material, two measurements were conducted and used for calculation of the average values and standard deviations.

### 3.2. Specimen Production Methods

**Figure 3:**
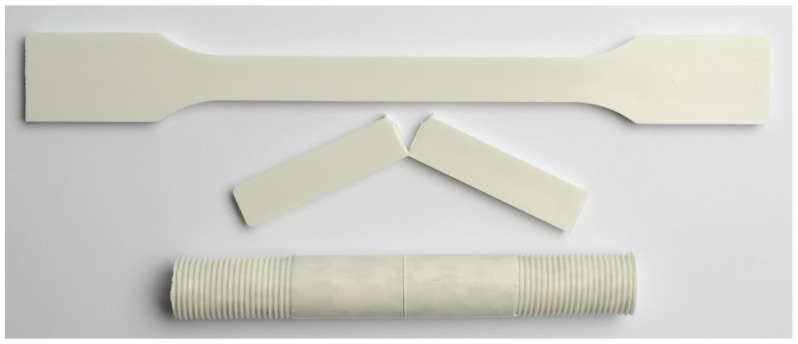
Image of the specimens used in this work made from the PE-HD recyclate rPE-A; from top down: a multipurpose specimen, a Charpy notched impact tested bar specimen, and a cracked round bar specimen.

According to ISO 17855-2 [27], tensile and impact test specimens for characterizing PE should be either compression or injection molded, depending on their MFR. Blow molding PE grades and, therefore, also all materials tested within this study have low enough MFRs to be compression molded. Nevertheless, for the sake of reproducibility, the authors chose to produce all multipurpose specimens (MPS) and bar specimens via injection molding according to ISO 294-1 [28], ISO 20753 [29], and ISO 17855-2 on a Victory 60 (Engel Austria GmbH, Schwertberg, Austria) with a 25 mm cylinder. As prescribed by ISO 17855-2, the melt temperature for injection molding of 210 °C and the injection velocity of 100 mm/s were used for all materials. The specimens were conditioned at 23 °C and 50% relative humidity for 3 days after production. After this conditioning, the MPS were used for tensile testing and the bar specimens were notched and used for Charpy notched impact testing in accordance with ISO 179-1 [30]. The Charpy type A notches, i.e., notches with a 0.25 mm notch radius, were produced with an RM2265 microtome (Leica Biosystems Nussloch GmbH, Nussloch, Germany) according to ISO 179-1 and measured with an Mitutoyo Absolute 547-313 digital thickness gauge with a wedge tip. An image of an MPS specimen together with a bar specimen are shown in Figure 3. The bar specimen is illustrated after failure to emphasize the induced notch and resulting crack plane.

For cracked round bar (CRB) specimen production according to ISO 18489 [21] for the ESCR experiments, plates of the size of 16 × 120 × 150 mm were compression molded in a specifically designed positive mold with the help of the hydraulic press Perfect Line (Langzauner GmbH, Lambrechten, Austria). Within the fully automated process, 280 g pellets were heated within the mold from room temperature to 180 °C with the weight of the mold on top of the material. An integrated temperature sensor allowed for direct measurement of the mold temperature. After the internal temperature of 180 °C was reached, it was held for 15 min. After that, the slow cooling with a cooling rate of 2 K/min was started. The full pressure of 10 MPa was applied to the material upon reaching a temperature of 135 °C during the cooldown step. Applying full pressure at higher temperatures leads to too much melt displacement. After reaching 40 °C, the pressure was released, the mold opened, and the plate manually removed. While ISO 18489 does not prescribe tempering of the produced plates, the authors observed conditioning influences, especially when testing at elevated temperatures. Therefore, the plates were cut into bars which then were tempered in accordance with a popular ESCR standard ISO 16770 [18] for 3 h at 100 °C and subsequently cooled down very slowly overnight in a Binder air oven (Binder GmbH, Tuttlingen, Germany). The tempered bars were then kept for at least 72 h at 23°C and 50% relative humidity for stress relaxation before being lathed on a lathe (EMCO GmbH, Hallein, Austria) to CRB specimens according to ISO 18489. A 0.3 mm thick industrial grade razor blade was used to notch the specimens. Before testing, the specimens were kept at 23 °C and 50% relative humidity for at least another 24 h after being notched. Directly before starting the test, the specimen was clamped 3 h in the test setup in the respective test medium at test temperature without any loading applied. A CRB specimen is shown in the bottom of Figure 3.

### 3.3. Differential Scanning Calorimetry Measurements

Differential scanning calorimetry (DSC) measurements were carried out on the differential scanning calorimeter DSC 8500 (PerkinElmer Inc., Waltham, MA, USA). Measurements were made according to ISO 11357-1 [31] and ISO 11357-3 [32]. Samples were cut from shoulders of injection molded MPS and from the middle section of bars from pressed plates before and after tempering and encapsulated in aluminum pans. The average sample weight was around 5 mg. Two different tests were performed. For the MPS from recyclates, the procedure consisted of an initial heating phase, subsequent cooling, and a second heating phase, each in the temperature range of 0 to 200 °C with a constant heating/cooling rate of 10 K/min with nitrogen as the purge gas and a flow rate of 20 mL/min. These measurements were accomplished to determine the melting peak in the second heating phase, which is characteristic for the semi-crystallinity achieved under controlled cooling in the DSC device. For the samples taken from the bars cut from pressed plates before and after tempering, only one heating run to determine the melting enthalpy change during tempering was performed with otherwise similar parameters. For each material, three samples, each cut from an individual MPS and bar, were used for the calculation of average values and standard deviations.

### 3.4. Oxidation Induction Temperature Measurements

A differential thermal analysis instrument from PerkinElmer, the DSC 4000, was utilized to characterize the oxidation induction temperature (dynamic OIT) according to ISO 11357-6 [33]. Samples were cut from shoulders of injection molded MPS and encapsuled in perforated aluminum pans. The average sample weight was around 8 mg. A single heating step between 23 and 300 °C was performed with a heating rate of 10 K/min and with synthetic air as purge gas with a flow rate of 20 mL/min. The point of intersect of the slope before oxidation and during oxidation gave the oxidation induction temperature in °C which gave an indication of the stabilization [34]. For each material, five samples, each cut from an individual MPS, were used for the calculation of average values and standard deviations.

### 3.5. Thermogravimetric Analyses

The thermogravimetric analysis (TGA) was performed on an STA 6000 simultaneous thermal analyzer (PerkinElmer, Waltham, MA, USA), according to ISO 11358-1 [35], using nitrogen as a purge gas with a flow rate of 20 mL/min. Samples of around 20 mg, usually one pellet, were put into open ceramic crucibles. The temperature program consisted only of one heating step from 30 to 850 °C with a heating rate of 20 K/min. For each material, two samples, each cut from an individual MPS, were used for the calculation of average values and standard deviations.

### 3.6. Tensile Tests

The tensile properties, tensile modulus, yield stress, and strain at break, were examined with a universal testing machine Zwick/Roell AllroundLine Z05 equipped with a Zwick/Roell multi-extensometer strain measurement system. Test parameters and MPS were used according to ISO 527-1 [16], ISO 527-2 [17], and ISO 17855-2 [27] with a testing speed of 1 mm/min for tensile modulus determination until a strain of 0.25% and after that 50 mm/min until failure. Calculations of tensile modulus, yield stress, and strain at break were carried out in accordance with ISO 527-1. Therefore, the tensile modulus was calculated as the slope of the stress/strain curve between 0.05 and 0.25% via regression. The yield stress was the stress at the first occurrence of strain increase without a stress increase and the strain at break was the strain when the specimen broke. The strain was recorded via a multi-extensometer until yield. From there, the nominal strain was calculated via Method B according to ISO 527-1 with the aid of the crosshead displacement. This process is integrated and automated in the used testing software TestXpert III (v1.61, ZwickRoell GmbH & Co. KG, Ulm, Germany). For each material, five MPS were tested for the calculation of average values and standard deviations.

### 3.7. Charpy Impact Tests

Impact properties were determined according to ISO 179-1 [30] on a Zwick/Roell HIT25P pendulum impact tester. After pretests to determine the suitable pendulum size (absorbed energy between 10 and 80% of the available energy at impact), a 2 Joule pendulum, which is the pendulum with the highest available energy that still conforms to these requirements, was chosen for testing all materials. Test conditions were 23 °C test temperature with type 1 specimen, edgewise blow direction, and notch type A, i.e., a 0.25 mm notch radius, or short ISO 179-1/1eA, which is one of the preferred methods of ISO 17855-2 [27]. For each material, ten specimens were tested for the calculation of average values and standard deviations.

### 3.8. Environmental Stress Cracking Experiments

As the authors have the experience and machinery to conduct cracked round bar fatigue crack-growth (FCG) experiments in harsh media conditions [36], the choice of measurement method was reached in favor of the cracked round bar method ISO 18489 [21]. The CRB specimens were tested with an electro-dynamic testing machine of the type Instron ElectroPuls E10000 (Illinois Tool Works Inc., Glenview, IL, USA). Sinusoidal loading profiles with a frequency of 10 Hz, an R-ratio of 0.1 and individually adjusted initial stress-intensity factor ranges (ΔK_I,ini_) were used to achieve testing times between 10 and 100 h. As testing environments, deionized water with 10 m% Igepal CO-630 (Rhodia S.A., La Défense, France) and air, both at 40 °C, were used. A specifically constructed testing chamber for environmentally superimposed CRB experiments, made from stainless steel and glass [22], was employed for containment of the media. For tests in the Igepal solution, a circulation pump was used to ensure the homogeneity of the surfactant mixture and the media temperature and also to flush out air bubbles from the crack. A fan mounted to the side of the specimen ensured a homogenic temperature within the testing chamber for the tests in air.

An in situ optical measurement of the crack length over the whole circumference of the specimen was utilized for investigations of crack growth [22]. With this, it was possible to calculate crack lengths during the measurement and link them via ΔK_I_ with the crack-growth rate. The ΔK_I_ value depends on the geometry, applied force range, and initial crack length as can be seen in the Equations (1)–(3) developed by Benthem and Koiter [37] and used within the CRB test standard ISO 18489. Hence, ΔK_I_ is a normalized value for the load affecting the crack tip.

(1) $$\Delta K_{I}=\frac{\Delta F}{\pi \cdot b^{2}}\cdot \sqrt{\frac{\pi \cdot a\cdot b}{r}}\cdot f\left(\frac{b}{r}\right)$$

(2) b=r−a

(3) $$f\left(\frac{b}{r}\right)=\frac{1}{2}\cdot \left[1+\frac{1}{2}\cdot \left(\frac{b}{r}\right)+\frac{3}{8}\cdot {\left(\frac{b}{r}\right)}^{2}-0.363\cdot {\left(\frac{b}{r}\right)}^{3}+0.731\cdot {\left(\frac{b}{r}\right)}^{4}\right]$$

where ΔK_I_ is the initial stress-intensity factor range in loading Mode I [38], ΔF the applied force range, a the measured crack length, r the radius of the specimen, b the ligament, and f(b/r) a geometry function.

Characteristic double logarithmic FCG kinetic curves were plotted providing the relation between the FCG rate, da/dN in mm/cycle, and the stress-intensity factor range, ΔK_I_ in MPa × m^0.5^. One CRB specimen was tested per material and medium.

## 4. Results

### 4.1. Melt Mass-Flow Rate

**Figure 4:**
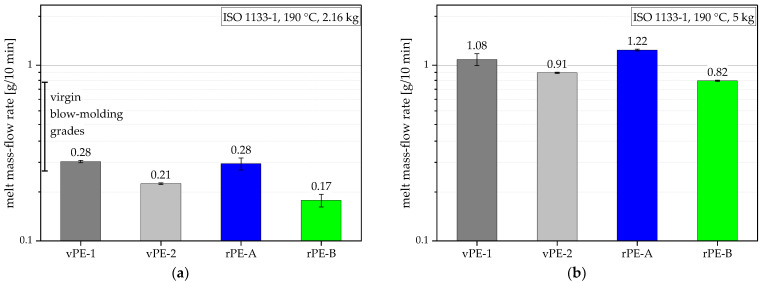
Graphical illustration and values of MFRs for all materials measured according to ISO 1133-1 at 190 °C and 2.16 kg (**a**) and 5 kg (**b**).

The MFRs for all materials are presented for 190 °C and 2.16 kg and for 190 °C and 5 kg in Figure 4a,b, respectively. The MFR values (190 °C and 2.16 kg) of vPE-1 and rPE-A fell within the MFR range for blow molding grades as depicted by the vertical bar in Figure 4a. vPE-2 and rPE-B showed values below these reference values. The low values of the latter materials could be either due to measurement inaccuracies or, especially for rPE-B, due to degradation-induced crosslinking [39,40,41]. The MFR measurements with both test weights exhibited similar trends. The 5 kg measurements are shown for comparison with recyclates, where often only 190 °C/5 kg values are provided in the datasheets.

### 4.2. Differential Scanning Calorimetry Measurements

**Figure 5:**
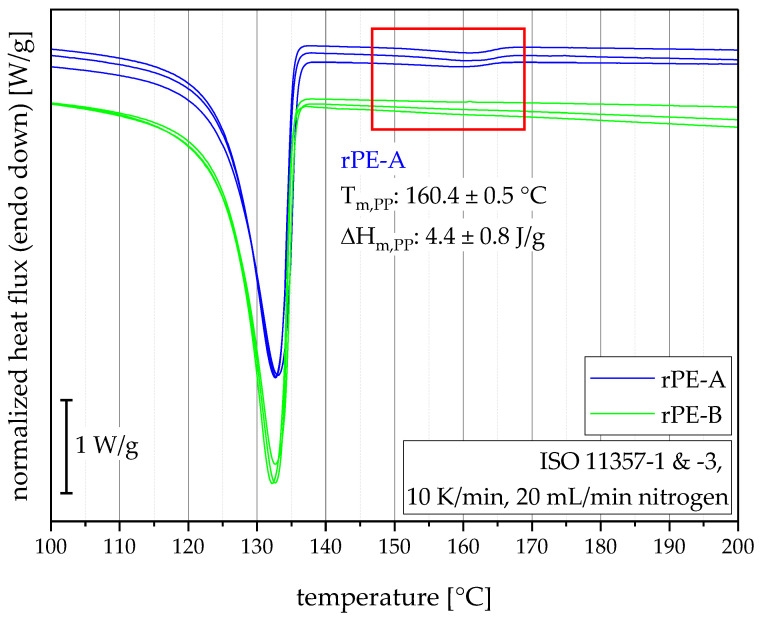
DSC thermograms of three repetition measurements for both recyclates and average values for the PP melting peak, which is framed by the red square, of the PE-HD recyclate rPE-A.

The DSC measurements on MPS specimens were only conducted for the recyclates with the goal to determine foreign polymer melting peaks. The thermograms were stacked so they could be looked at individually and they show that all three samples from three individual MPS exhibited comparable results, as illustrated in Figure 5. When looking at the area between 150 °C and 170 °C, a small endothermic peak can be seen in the thermograms of rPE-A, which suggests polypropylene (PP) contamination, most likely due to bad separation during the recycling process, for which such cross-contamination in polyolefins can be often seen in polyolefin recycling [10,42,43]. rPE-B did not show any other endothermic peak apart from the polyethylene (PE) melting peak. Hence, according to DSC, it was free of discernable PP contamination.

The DSC measurements of samples cut from the bars which are used for the manufacturing of CRB specimens were conducted to determine the melting enthalpy of the PE phase. Contrary to the measurements conducted on samples taken from MPS, the first heating run is shown instead of the second heating run. In this way, the effect of the tempering on the materials morphology could be investigated. The PP melting enthalpy is not shown for these tests as the low sample volume and missing homogenization step (injection molding) leads to high scattering of the quantifiable PP melting enthalpy in rPE-A. In comparison, the homogenization by injection molding of MPS distributes the PP contamination to each DSC sample, hence creates, despite the small sample volume, appropriate specimens for detecting trace amounts of contamination.

**Figure 6:**
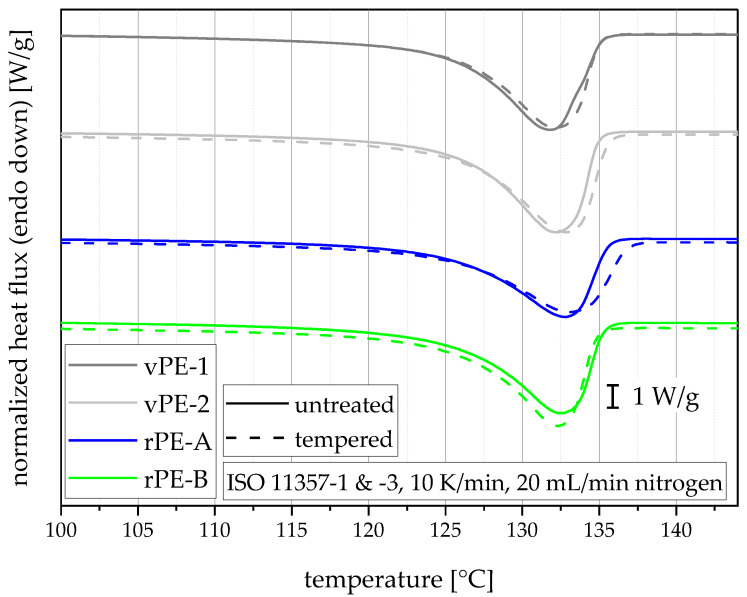
Averaged DSC thermograms (three measurements for one curve) from samples cut from untreated and tempered bars used to produce CRB specimens. Curves are stacked for better visibility.

While there are no significant differences in the melting temperatures, all materials showed a distinct rise in PE melting enthalpy due to the tempering process, as can be seen in the thermograms shown in Figure 6 and values in Table 1. Furthermore, most materials showed a distinct decrease in standard deviation, indicating a homogenization of crystal structures. The increases of the melting enthalpies translate to rises in the degree of crystallinity of 62 to 65% (rPE-A), 70 to 71% (rPE-B), 73 to 74% (vPE-1), and 73 to 77% (vPE-2) [44], which all lie within the PE-HD range of 60–80% [10]. 

### 4.3. Oxidation Induction Temperatures

A higher dynamic OIT shows a higher resistance to thermo-oxidative degradation and can be used as an indicator for the effectiveness of stabilizers incorporated into the material as they affect the material’s vulnerability to harsh oxidative environments [45]. Furthermore, the stabilization influences the localized aging of the crack tip [46,47] and, therefore, should influence the ESCR.

**Figure 7:**
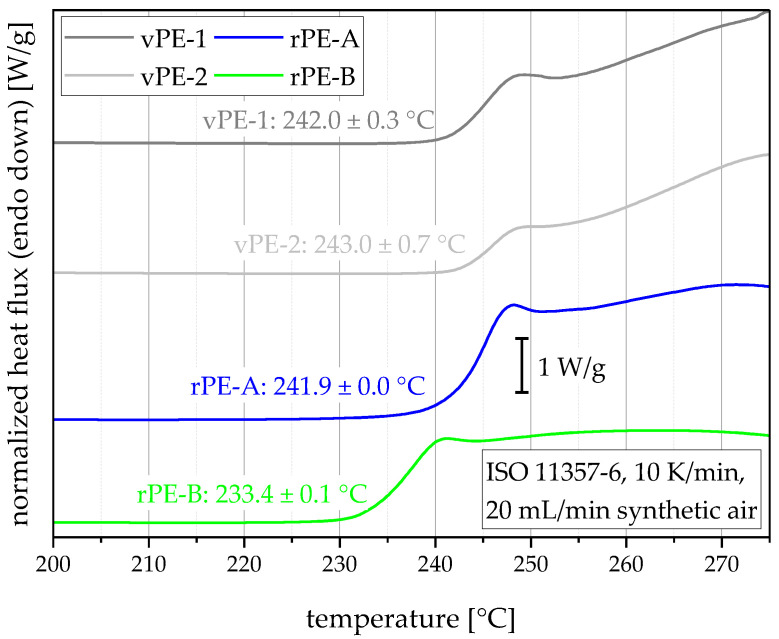
Averaged DSC thermograms (two or three measurements for one curve) and values of oxidation induction temperatures for all materials. Curves are stacked for better visibility.

The DSC thermograms in synthetic air together with the calculated dynamic OITs are shown in Figure 7. Both virgin polymers showed high dynamic OIT values of 241.9 °C (vPE-1) and 243.0 °C (vPE-2). rPE-A surprises with a dynamic OIT value of 241.9 °C, suggesting that the recyclate produced added stabilizers during the recyclate production. The second recyclate rPE-B showed a lower dynamic OIT value of 233.4 °C. Therefore, vPE-1, vPE-2, and rPE-A should show a considerably higher resistance against harsh environments than rPE-B in terms of thermo-oxidative degradation.

### 4.4. Thermogravimetric Analyses

**Figure 8:**
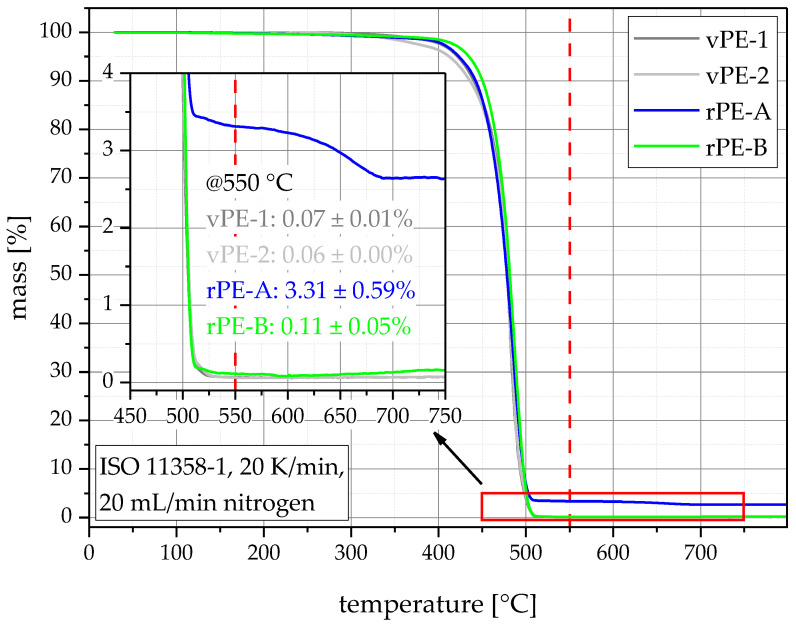
Thermograms of the TGA of all materials with average values and sample standard deviations of the pyrolysis residue at 550 °C (indicated by the vertical red dashed line).

TGA was used to show the inorganic contamination level of the materials. Unfilled polyolefins are expected to show no pyrolysis residue [44]. rPE-A, therefore, shows the distinctive signs of contamination, as its pyrolysis residue at 550 °C lies around 3.31%. rPE-B contains only very low amounts of inorganic contamination with a pyrolysis residue of 0.11%. The virgin polymers vPE-1 and vPE-2 showed even smaller pyrolysis residues of 0.07% (vPE-1) and 0.06% (vPE-2). The zoomed in section in Figure 8 shows the mass change between 450 and 750 °C, and reveals a second decomposition step for rPE-A at around 650 °C. This step can be attributed to the cleavage of CO_2_ from CaCO_3_ which would also explain the white color of rPE-A [10].

### 4.5. Tensile Properties

**Figure 9:**
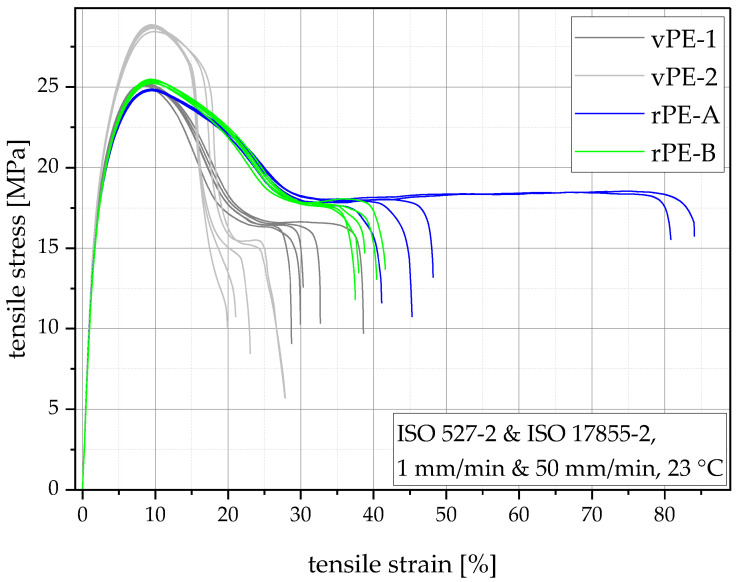
Tensile stress vs. tensile strain diagram showing five measurements of each material.

Tensile tests are a good and quick measure to gain an overview of the mechanical performance of materials. Recyclers seldom have the machinery to produce compression molded specimens for tensile or Charpy tests, hence, in their datasheets, they depict values from injection molded specimens. There is a discrepancy in the mechanical parameters between differently produced specimens with higher values usually for compression molded specimens [48], at least for the tensile properties. Virgin PE-HD producers are aware of the discrepancy and, therefore, present values from specimens produced in compression molding in their datasheets. This should be kept in mind when comparing the values from Table 2 with datasheet values from PE-HD suppliers. Tensile curves are provided in Figure 9.

The tensile moduli show a clear distinction between the virgin and recycled PE-HD grades. Both virgin grades show higher tensile moduli (vPE-1 with 977 MPa and vPE-2 with 1020 MPa) than the two recyclates (rPE-A with 939 MPa and rPE-B with 914 MPa). The yield stresses show a different trend. vPE-1 (25.2 MPa), rPE-A (24.8 MPa) and rPE-B (25.3 MPa) show similar yield stresses while vPE-2 exceeds them with a very high value of 28.7 MPa. The strain at break values mimic the MFR trend shown in Figure 4. This happens due to the shear-induced orientation of the polymer chains during injection molding. Materials with higher MFR experience less shear, hence are less oriented, and show higher strain at break values [49]. At this low strain at break values, clean polymers usually do not show a high standard deviation within strain at break. As rPE-A shows a higher level of contamination than rPE-B, as shown in Figure 5 and Figure 8, for rPE-A, it is more likely to have random occurring contamination agglomerates. These agglomerates can act as crack initiators and lead to premature failure of the specimen, which subsequently can lead to a high standard deviation in strain at break values [50]. This can also be seen in Figure 9, which shows that three out of five samples from rPE-A showed low strain at break values of around 45% while two samples had very long strain at break values of above 80%.

While the measured properties cannot be directly compared to the datasheet values of virgin grades due to their different manufacturing process (injection molded instead of compression molded), the comparison of the virgin blow molding grades to the recyclates which were produced with the same parameters show that the recyclates investigated lie within the range of virgin PE-HD blow molding grades.

### 4.6. Charpy Notched Impact Strengths

The impact strength can be measured according to several standardized methods with varying loading principles, geometries, and testing temperatures. One of the most used methods in Europe, as can be seen in summary datasheets of European suppliers [12,15], is the Charpy notched impact strength measurement. The impact strength is a crucial property for blow molded PE bottles as its value depicts the ability of a bottle/container to survive a fall without critical failure or breaking/tearing of the wall.

The Charpy notched impact strengths of all materials are depicted in Table 2. Both virgin PEs show significantly higher values than the two recyclates. rPE-2 had the highest value of 46.6 kJ/m^2^, followed by vPE-1 with 35.9 kJ/m^2^, rPE-B with 26.8 kJ/m^2^, and finally rPE-A with 23.5 kJ/m^2^. The lower values of the recyclates, especially of rPE-A, could again be explained by contamination within the polymer matrix [8,10], as also shown in Figure 5 and Figure 8, which can significantly lower the Charpy notched impact strength [10]. On average, the recyclates showed 30% lower Charpy notched impact strength compared to vPE-1. This could be a problem for critical-use cases and must be considered during the development of the recyclate packaging geometry and/or the wall thickness used for packaging.

### 4.7. Environmental Stress Cracking Resistances

The ESCR is a combination of two superimposed damage mechanisms, slow crack growth due to mechanical loading (usually static) and environmentally (both media and temperature) induced acceleration of that slow crack growth. The testing method presented within this paper is a first approach to combine a customary ESCR-testing medium (Igepal CO-630, from ASTM D1693 [19] and D2561 [20]) with the cyclic loading cracked round bar test method according to ISO 18489 [21].

**Figure 10:**
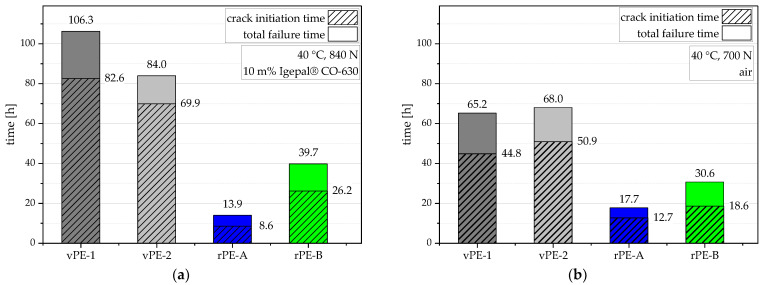
Graphical illustration and values of crack initiation times and total failure times at 40 °C in a solution of deionized water and 10 m% Igepal CO-630 (**a**) and in air (**b**).

**Figure 11:**
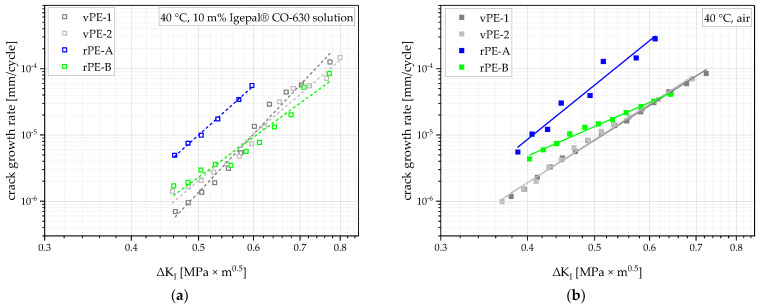
Crack-growth kinetics tested at 40 °C for all materials in a solution of deionized water with 10 m% Igepal CO-630 (**a**) and in air (**b**). Linear fits highlight the stable stress-intensity factor range dependent on crack growth.

Test results are shown in two ways. Figure 10 illustrates the crack initiation time as well as the total failure time which was determined with the help of an optical crack length measurement system for measurements [22] in a solution of deionized water with 10 m% Igepal CO-630 (Figure 10a) and in air (Figure 10b), both at 40 °C. Figure 11 shows the crack-growth kinetic curves of these measurements depicting the actual crack-growth rate within the material without the crack initiation time where no crack growth occurs.

Figure 10a (40 °C, 840 N maximum force, 10 m% Igepal solution) illustrates that both virgin grades exhibit much higher testing times compared to the recyclates. vPE-1 shows the highest value of 106.4 h followed by 84.0 h for vPE-2. rPE-B indicates with its 39.7 h an almost three times higher testing time than rPE-A with 13.9 h. Both crack initiation times and failure times in air are lower than in the Igepal solution for all materials except for rPE-A, although they were tested at higher maximum forces (840 N vs. 700 N), as shown in Figure 10b (40 °C, 700 N maximum force, air). Both virgin grades vPE-1 and vPE-2 exhibited similar crack initiation times of 44.8 h (vPE-1) and 50.9 h (vPE-2) and total failure times of 65.2 h (vPE-1) and 68.0 h (vPE-2) in air.

The stress-intensity factor range (ΔK_I_)-dependent crack-growth rate development in the solution of deionized water with 10 m% Igepal CO-630 is shown in Figure 11. As the force range applied to the specimen stayed the same during the whole measurement, the only variable contributing to the change in ΔK_I_ was the crack length. Hence, each measurement started at the bottom (low crack-growth rate) left (low crack length, or rather ΔK_I_). As the crack propagated, ΔK_I_ and the crack-growth rate rose. vPE-1, vPE-2 and rPE-B showed crack-growth rates of similar magnitude while rPE-A showed an around five times higher crack-growth rate at a similar ΔK_I_. Compared to vPE-1, vPE-2 and rPE-B showed much higher crack-growth rates at lower ΔK_I_ values, which explains the much higher crack-growth time (total failure time minus crack initiation time, shown in Figure 10a) of vPE-1.

The crack-growth kinetics of the measurements in air, which are shown in Figure 11b, altogether move to the left and up, indicating faster crack-growth rates at a similar ΔK_I_. vPE-1 and vPE-2 are indistinguishable from each other, indicating a similar slow crack-growth resistance. rPE-B shows distinctly higher crack-growth rates at low ΔK_I_ values, significantly shortening its crack-growth time compared to the virgin PEs. rPE-A again shows the highest crack-growth rate of all materials, revealing its low resistance to slow crack growth.

## 5. Discussion

By combining the findings of the results section, the following statements concerning the performance of both PE-HD recyclates can be made: the MFRs of the investigated recyclates suggest a feedstock of blow-molded PE-HD product waste. rPE-B shows the lower MFR of both recyclates. Both the DSC (see Figure 5) and the TGA (see Figure 8) measurements resulted in a clear distinction of rPE-A as the more contaminated and rPE-B as the cleaner recyclate. The oxidation induction temperatures (dynamic OIT) showed a different trend. Here, rPE-A showed the higher dynamic OIT, hence it should be more stable against thermo-oxidative degradation. The tensile and impact performances of both recyclates were very similar. The results of the ESCR measurements in the solution of deionized water with 10 m% Igepal CO-630 showed a clear distinction between rPE-A and rPE-B in Figure 10a and Figure 11a. It is therefore safe to conclude, that rPE-B had the better resistance against slow crack growth in this environment, or rather had the higher ESCR. While the dynamic OIT measurements revealed an oxidative resistance of rPE-A, the lower contamination level exhibited by DSC and TGA as well as the lower MFR indicated a better crack-growth performance for rPE-B [49].

**Figure 12:**
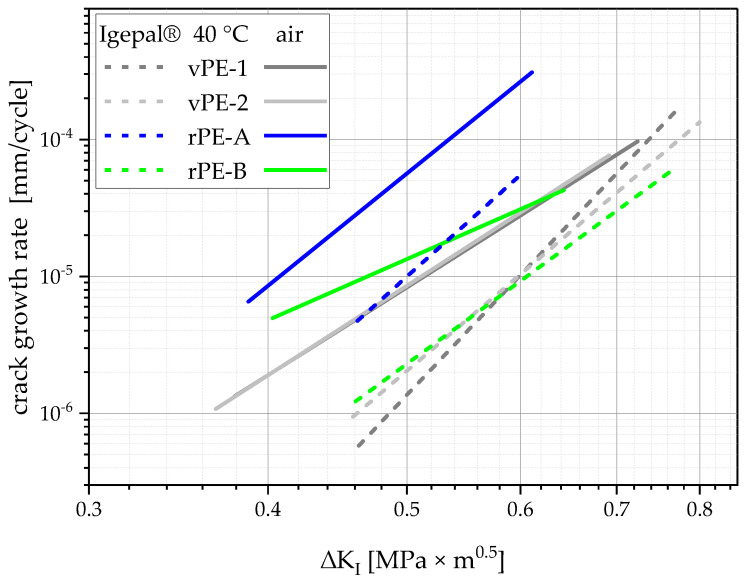
Linear fits of crack-growth kinetics tested at 40 °C for all materials in a solution of deionized water and 10 m% Igepal CO-630 (dashed lines) and in air (full lines).

The cracked round bar experiments at 40 °C in air were conducted for investigation of the effect of Igepal CO-630 on the testing time and crack-growth kinetics. A comparison of both environments can be seen in Figure 12. Here, it is clear that all materials performed better or rather had a higher resistance to slow crack growth in the Igepal solution. This effect has been proven before [51,52]. Hence, the combination of both measures, cyclic loading and Igepal CO-630 which each alone would accelerate crack growth, shows an opposed effect by decelerating crack growth. Nevertheless, conclusions can be drawn from the results in both media. When comparing crack-growth kinetics in Igepal solution and in air, one result that stands out is the change in crack-growth rate of rPE-B at low ΔK_I_ values. The crack-growth rate at these low ΔK_I_ values marked the beginning of crack growth directly after the crack initiation time. In this case, the crack propagated in the plastic zone which was exposed to the environment during the whole crack initiation time. In contrast, higher ΔK_I_ values showed crack growth at higher crack lengths, that is, plastic zones which were not exposed to the environment during crack initiation. The flatter slope of rPE-B in the air measurement could, therefore, indicate that rPE-B was significantly more damaged during crack initiation in air than the other materials, which could also be explained by its lower dynamic OIT value, as seen in Figure 7. This different behavior in the two media is also true for vPE-1 and vPE-2, although less extreme. Therefore, the higher stabilization within the virgin plastics has a positive effect against this local crack tip aging. For rPE-A, almost no change in slope was visible, which could be explained by its generally short crack initiation time (as seen in Figure 10) and the high dynamic OIT (as seen in Figure 7).

These above-drawn conclusions imply that 40 °C warm air induces more local aging in PE than 40 °C Igepal solution. The availability and mobility of oxygen is certainly higher in air than in the liquid surfactant mixture. Moreover, the crack-growth-accelerating effect of Igepal CO-630, elaborated upon in the literature, can be attributed to its reduction of the “frictional resistance to chain slippage” [52], and not to local oxidative aging as other environments [46], which also corroborates the above-drawn conclusion.

## 6. Conclusions

With the presented properties of the various tested PE-HD virgin blow molding grades and recyclates, the applicability of recyclates for blow molding applications can only be estimated in terms of their mechanical properties. The processability with existing blow molding processes was not investigated in this paper and should also be considered when it comes to replacing virgin materials with recyclates. While the short-term mechanical properties measured by tensile and impact tests show promising results, the resistance to slow crack growth and the ESCR of recyclates may be much worse compared to virgin materials. The effect of Igepal CO-630 on the ESCR of recyclates complicates the evaluation and interpretation of the results. While the crack-growth kinetics of the recyclate rPE-B and the virgin grades look comparable, the testing time and, here specifically, the crack initiation time for the recyclate was much shorter. Shorter testing times could also be expected for other ESCR methods where only testing time and not crack-growth rate is investigated. It can be assumed that contamination found within recyclates contributes to an accelerated crack initiation and therefore they are essential for the materials’ ESCR performance. This effect could be verified by future testing on intentionally contaminated PE-HD virgin grades.

## Figures and Tables

**Table 1:** PE melting temperatures and enthalpies for untreated and tempered bars cut from pressed plates intended for CRB specimen production.

	vPE-1		vPE-2		rPE-A		rPE-B	
**PE melting temperature** **(untreated) [°C]**	132.0	±0.8	132.1	±0.3	132.8	±0.2	132.5	±0.3
**PE melting temperature** **(tempered) [°C]**	132.2	±0.2	133.1	±0.3	133.0	±0.2	132.3	±0.2
**PE melting enthalpy** **(untreated) [J/g]**	212.9	±1.9	213.6	±2.4	181.3	±3.8	204.3	±3.0
**PE melting enthalpy** **(tempered) [J/g]**	215.9	±0.8	224.5	±1.8	190.9	±4.3	206.7	±0.2

**Table 2:** Tensile and Charpy notched impact properties (average values and sample standard deviations).

	vPE-1		vPE-2		rPE-A		rPE-B	
**Tensile modulus [MPa]**	977	±7.2	1020	±5.6	939	±3.1	914	±2.9
**Yield stress [MPa]**	25.2	±0.1	28.7	±0.2	24.8	±0.0	25.3	±0.1
**Strain at yield [%]**	8.6	±0.2	9.6	±0.2	9.5	±0.2	9.6	±0.1
**Strain at break [%]**	31.7	±3.6	23.9	±3.7	59.9	±20.8	39.3	±1.7
**Charpy notched impact strength [kJ/m^2^]**	35.9	±0.8	46.6	±6.3	23.5	±3.0	26.8	±1.6
